# Effect of Combined Therapy of Virtual Reality and Transcranial Direct Current Stimulation in Children and Adolescents With Cerebral Palsy: A Study Protocol for a Triple-Blinded Randomized Controlled Crossover Trial

**DOI:** 10.3389/fneur.2020.00953

**Published:** 2020-09-02

**Authors:** Talita Dias da Silva, Anne Michelli Gomes Gonçalves Fontes, Barbara Soares de Oliveira-Furlan, Tatiane Tedeschi Roque, Ana Izabel Izidório Lima, Bruna Mayara Magalhães de Souza, Camila Aparecida de Oliveira Alberissi, Ana Clara Silveira, Íbis Ariana Peña de Moraes, Johnny Collett, Roger Pereira Silva, Marina Junqueira Airoldi, Denise Cardoso Ribeiro-Papa, Helen Dawes, Carlos Bandeira de Mello Monteiro

**Affiliations:** ^1^Programa de Pós-Graduação em Ciências da Reabilitação, Faculdade de Medicina da Universidade de São Paulo (FMUSP), São Paulo, Brazil; ^2^Departamento de Medicina (Cardiologia), Universidade Federal de São Paulo (UNIFESP), São Paulo, Brazil; ^3^Grupo de Pesquisa e Aplicações Tecnológicas em Reabilitação (PATER), Escola de Artes, Ciências e Humanidades, Universidade de São Paulo (EACH-USP), São Paulo, Brazil; ^4^Faculdade de Medicina, Universidade Cidade de São Paulo (UNICID), São Paulo, Brazil; ^5^Institute of Nursing and Allied Health Research, Oxford Brookes University, Oxford, United Kingdom; ^6^Department of Clinical Neurology, University of Oxford, Oxford, United Kingdom

**Keywords:** cerebral palsy, virtual reality exposure therapy, plasticity, motor rehabilitation, autonomic nervous system, non-invasive brain stimulation, transcranial direct current stimulation

## Abstract

**Background:** Transcranial direct current stimulation (tDCS) and therapy-based virtual reality (VR) have been investigated separately. They have shown promise as efficient and engaging new tools in the neurological rehabilitation of individuals with cerebral palsy (CP). However, the recent literature encourages investigation of the combination of therapy tools in order to potentiate clinic effects and its mechanisms.

**Methods:** A triple-blinded randomised sham-controlled crossover trial will be performed. Thirty-six individuals with gross motor function of levels I to IV (aged 4–14 years old) will be recruited. Individuals will be randomly assigned to Group A (active first) or S (sham first): Group A will start with ten sessions of active tDSC combined with VR tasks. After a 1-month washout, this group will be reallocated to another ten sessions with sham tDCS combined with VR tasks. In contrast, Group S will carry out the opposite protocol, starting with sham tDCS. For the active tDCS the protocol will use low frequency tDCS [intensity of 1 milliampere (mA)] over the primary cortex (M1) area on the dominant side of the brain. Clinical evaluations (reaction times and coincident timing through VR, functional scales: Abilhand-Kids, ACTIVLIM-CP, Paediatric Evaluation of Disability Inventory-PEDI- and heart rate variability-HRV) will be performed at baseline, during, and after active and sham tDCS.

**Conclusion:** tDCS has produced positive results in treating individuals with CP; thus, its combination with new technologies shows promise as a potential mechanism for improving neurological functioning. The results of this study may provide new insights into motor rehabilitation, thereby contributing to the better use of combined tDCS and VR in people with CP.

**Trial Registration:** ClinicalTrials.gov, NCT04044677. Registered on 05 August 2019.

## Introduction

Cerebral palsy (CP) describes a group of permanent disorders of movement and posture that limit activity. It is attributed to non-progressive disturbances that occur in the developing foetal or infant brain (1). The difficulties that accompany individuals with CP lead to their registration in different and continuous rehabilitation programmes to promote the development of general motor skills, and some studies defend the importance of upper limb tasks to promote physical activity for people with CP. According to Pontén et al. (2) and Sarcher et al. (3), contractions in the upper limbs of individuals with CP start early and require adequate intervention and special attention to provide increases in (or maintenance of) range of movement, better performance and physical activity (especially for the ones with less global mobility), improving the performance of the functions of daily life, increasing independence, activities, and participation (4). Likewise, there is growing evidence of the higher prevalence of metabolic syndrome, cardiovascular disease risk factors, and autonomic nervous system (ANS) dysfunctions in adults with CP (5). According to Katz-Leurer and Amichai (6), because of the sedentary behaviour that results from their limited mobility (i.e., the more limited the mobility, the less activity), individuals with CP are more disposed to chronic disorders such as heart conditions and hypertension.

Thus, considering the presence of musculoskeletal and metabolic conditions in individuals with CP, professionals involved in their care need to consider all the impaired structures and functions and look for proposals for interventions based on scientific evidence that can effectively and comprehensively treat the limitations and restrictions caused by the brain injury (7). To do so, they rely on modern technologies to create new practices and interventions to stimulate different body structures and physiological responses, even for those with more severe conditions, to optimise the acquisition of mo(tor skills, which leads to a more active life (8, 9).

In addition to evidence for the benefits of different techniques for the treatment in rehabilitation of individuals with CP (8–10), recent studies encourage the combination of interventions and technologies as a promising approach for rehabilitation (11). Currently, few studies had investigated the effect of combined therapies in the rehabilitation of individuals with CP, though they presented some encouraging results (12–15). Muszkat et al. (11), suggested that the combination of therapeutic tools should be encouraged to enhance clinical effects and provide more effective and long-lasting results. In this sense, with the increasing accessibility and evolution of technology, virtual reality (VR) and transcranial direct current stimulation (tDCS) have the potential to advance the treatment of CP (10).

The use of VR in rehabilitation is a modern concept of treatment that is based on the use of games and tasks in virtual environments to stimulate physical and cognitive functions in individuals with different types of deficiencies (16, 17). In VR, the user interacts with a three-dimensional environment through remote input devices, such as a keyboard or a mouse (a non-immersive environment), or by more advanced devices (an immersive environment) such as a camera, glasses or special gloves (16). Some studies were carried out using VR in individuals with CP, and the effects were significantly positive concerning the balance and strength of lower limbs (18), learning of general motor skills (19), day-to-day activities (19), and improvement of general learning processes with increased attention in the task (20).

Transcranial direct current stimulation is a non-invasive neuromodulatory technique that produces benefits in the sensorimotor and physiological functions of individuals with different neurological deficits (21), including individuals with CP [see the review by (22)]. The tDCS uses low electrical current (1–2 mA) to modulate the resting potential of neurons below the stimulated site (23). The action mechanism of tDCS is related to the changes in the rates of spontaneous neuronal firing and synaptic and non-synaptic plasticity, whichh influences changes in the resting polarisation of the neurons, and promotes neuroplasticity in cortical areas critically involved in the performance of tasks and in promoting functional benefits (24).

The benefits of tDCS include the flexibility to use it for different activities and exercises (as it presents a mobile characteristic) and the possibility of combining it with other interventions. Spampinato et al. (25) showed that the combination of tDCS with a task using reward characteristics produced neurophysiological modulation of inhibitory networks, and it resulted in enhanced retention of the learned task. Thus, it can be used during fine motor tasks [to reinforce learning of coordinative tasks; (26)], global movements [to increase range of movement; (22)] and physical activities [in order to facilitate motor activities; (12)], and to improve heart and autonomic conditions (27).

Some studies have produced positive results when combining VR and tDCS therapies in stimulating the lower limbs for gait improvement (12) and balance (28, 29). However, no studies have investigated VR and tDCS interventions using an upper limb motor task, which might benefit different clinical conditions of individuals with CP. We organised a triple-blinded, randomised, and controlled crossover trial to investigate the upper limb motor function of individuals with CP with the aims of (1) investigating the effectiveness of the use of tDCS while performing a non-immersive VR task on upper limb motor function and (2) to analysing the influence of a combined VR and tDCS in upper limb motor function through different functional assessment scales, reaction times, and coincident timing analysis, as well as physiological analyses such as heart rate variability.

We hypothesise that all individuals with CP will show an improvement in performance after practising a non-immersive virtual reality task, with benefits in upper limb functional scales, reaction times and, coincident timing analysis underpinned by an adaptation of autonomic neural physiological control after the protocol, and retention of these variables at the 30-day follow-up. However, such improvement and benefits will be more evident after the application of active tDCS than the sham (placebo) tDCS group. If this hypothesis is confirmed, the results of this study will be relevant to the treatment of individuals with CP.

## Methods/Design

**Figure 1:**
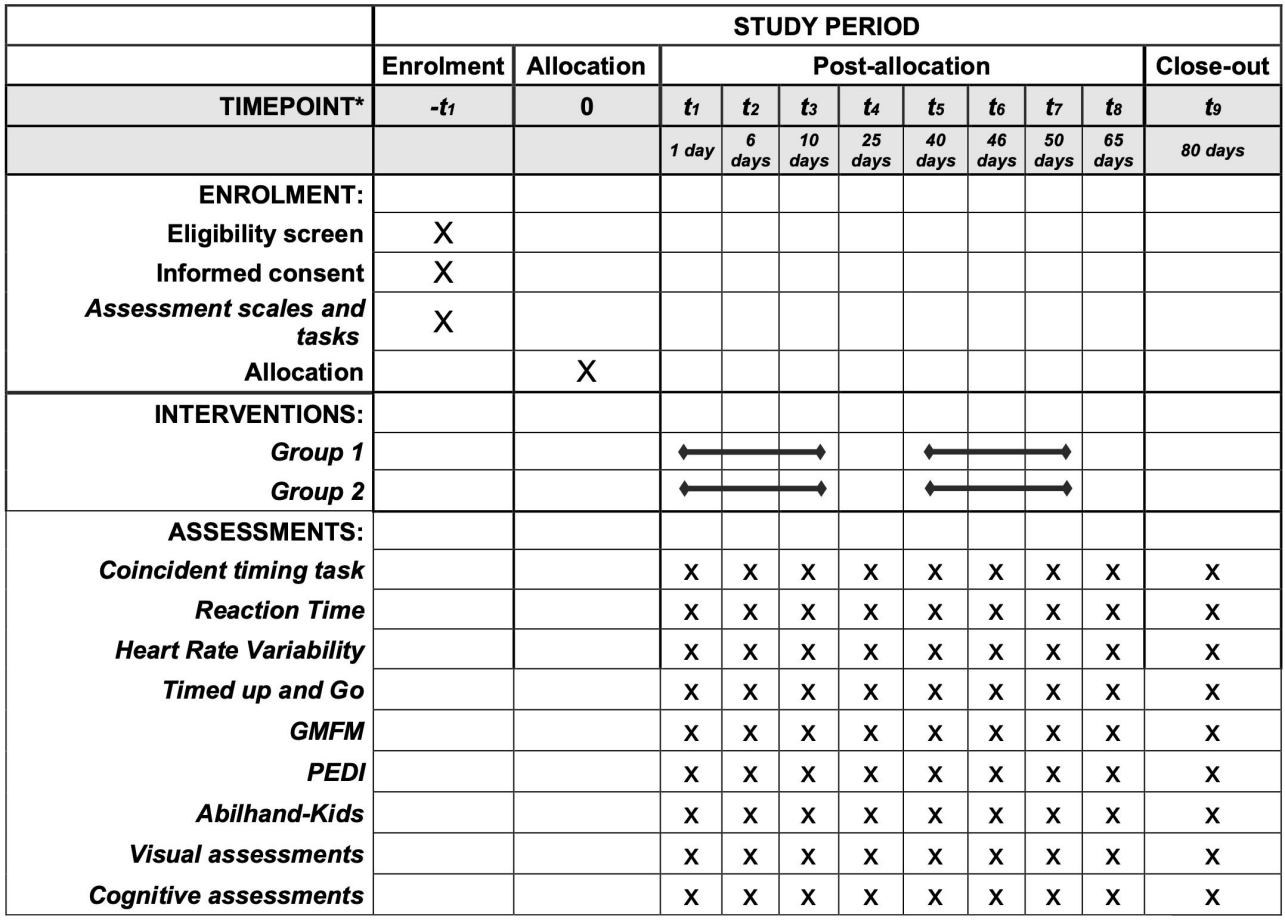
SPIRIT: Description of the study protocol, schedule of enrolment, interventions, and assessments. ^*^List of specific timepoints in this row.

We registered this trial on ClinicalTrials.gov (NCT04044677). This paper has been reported in accordance with the Standard Protocol Items: Recommendations for Interventional Trials (SPIRIT) (30) (Figure 1 and Table 1).

**Table 1:** Trial characteristics based on WHO Trial Registration Data Set.

**Data category**	**Trial information**
Primary registry and trial identifying number	ClinicalTrials.gov, ID: NCT04044677
Date of registration in primary registry	05 August 2019
Secondary identifying numbers	Ethical Committee of the University of São Paulo, under the number CAAE: 99577318.0.0000.5390
Source(s) of monetary or material support	Coordenação de Aperfeiçoamento de Pessoal de Nível Superior–Brasil (CAPES)
Primary sponsor	University of São Paulo–USP
Secondary sponsor(s)	NA
Contact for public queries	TDS, CBMM
Contact for scientific queries	TDS, CBMM
Public title	Virtual reality therapy and transcranial direct current stimulation in cerebral palsy
Scientific title	Effect of combined virtual reality therapy and transcranial direct current stimulation on children and adolescents with cerebral palsy
Country of recruitment	Brazil
Health condition(s) or problem(s) studied	Cerebral palsy
Interventions	Group 1 will start with 10 sessions of active tDSC combined with VR tasks. After a 1 month washout, this group will be reallocated to another 10 sessions with sham tDCS combined with VR tasks. Meanwhile, group 2 will carry out the opposite protocol (i.e., participants will start an allocated 10 sessions of sham TDCS combined with VR tasks, and after a 1 month washout period will be reallocated to 10 sessions of active tDCS combined with VR tasks).
Key inclusion and exclusion criteria	Inclusion criteria: the agreement to participate in the research from themselves and their legal guardians; a clinical diagnosis of CP will be performed by a neuropaediatric clinician; with Gross Motor Function Classification System (GMFCS) levels I to IV; and Manual Ability Classification System (MACS) I to IV; age range 4–14 years. Exclusion criteria: do not understand the tasks; motor difficulties that impede the completing of the virtual tasks; cardiac diseases that impede the assessment of heart rate variability (HRV) and surgery; use of an upper limb spasticity inhibitor during the previous 6 months; metal prosthesis on the head. Withdrawal criteria: participants will be withdrawn from the study if they are not willing to continue cannot be present on the day of the experiment, or miss two treatment sessions.
Study type interventional allocation	Randomised
Masking	Triple-blinded
Assignment	Crossover
Primary purpose	Treatment
Date of first enrolment	March 2019
Target sample size	35
Recruitment status	Recruiting
Primary outcome(s)	Motor skills improvement
Key secondary outcome(s)	HRV improvement

### Overview of the Study Design

**Figure 2:**
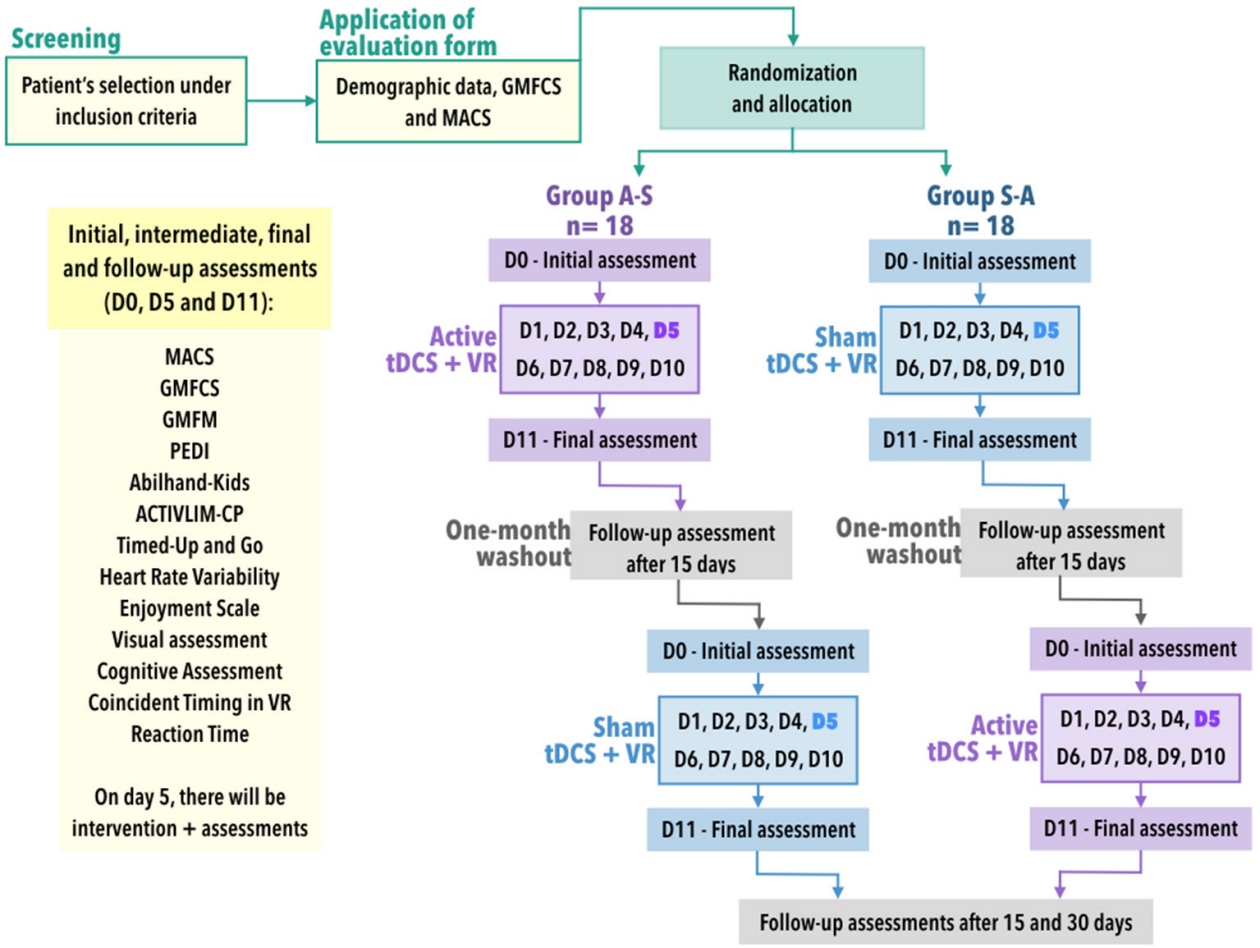
Study design. MACS, Manual Ability Classification System; GMFCS, Gross Motor Function Classification System; PEDI, Pediatric Evaluation of Disability Inventory; VR, virtual reality; n: sample size; tDCS, transcranial direct current stimulation; D0-D11, days of interventions in which D0 is the baseline, D1 to D10 are the days of interventions and the D11 is the day of the final assessments. Participants and sampling.

A triple-blinded randomised controlled crossover trial with a 1:1 allocation ratio will be conducted, and all participants will undertake non-immersive VR tasks and active or sham tDCS. Groups A–S will start with 10 daily sessions of tDCS-active combined with VR tasks for 20 min. After a 1 month washout, this group will be reallocated to another 10 daily sessions of 20 min with sham tDCS combined with VR tasks. Meanwhile, groups S–A will carry out the opposite protocol (participants will start an allocated 10 sessions of sham tDCS combined with VR tasks, and after a 1 month washout period will be reallocated to 10 sessions of active tDCS combined with VR tasks). The 1 month washout period has been used and was shown to be sufficient to reset the effects of the first 10 sessions in Biabani et al. (31). Figure 2 summarises the planned experimental design. This research protocol follows the SPIRIT recommendations.

Thirty-six participants will be recruited through referral by the coordinators of three clinics in Brazil: *Intensiva, Intertherapy*, and *Therapies*, located in São Paulo state. Those interested in participating will undergo a detailed screening using the eligibility criteria for enrolment in the study.

The sample size was calculated using statistical software (GPower 3.1.5) on the main outcome measure (i.e., the motor score). This calculation was based on data from one study with a group of individuals with CP who received tDCS (32). The power was 0.80; the alpha was 0.05; and the effect size was 0.65 (Cohen's d). The sample estimation indicated that 28 participants would be necessary (i.e., 14 per group). With an adjustment to allow for a withdrawal rate (20%), we will recruit 36 participants.

### Inclusion Criteria

Participants will be included if they have: the agreement to participate in the research from themselves [by signing assent form (33)] and their legal guardians (by signing a consent form); a clinical diagnosis of CP will be carried out by a neuropaediatric clinician; with GMFCS levels I to IV and MACS I to IV; age ranging from 4 to 14 years.

### Exclusion Criteria

Participants will be excluded if they (1) do not understand the tasks—the understanding of the task will be evaluated through five attempts at each task in VR, because even with a low intelligence quotient (IQ) a large number of the children and adolescents can understand virtual tasks and interact with improved performance; (2) motor difficulties that impede the completing of the virtual tasks; (3) cardiac diseases that impede the assessment of HRV; (4) surgery or use of an upper limb spasticity inhibitor during the last 6 months; and (5) a metal prosthesis in the head.

### Withdrawal Criteria

Participants will be withdrawn from the study if they are not willing to continue, cannot be present on the day of the experiment, or miss two treatment sessions out of 10 (four in total).

### Randomisation

Participants will be randomly allocated to either group A-S (active tDCS and VR tasks) or group S-A (sham tDCS and VR tasks) with a 1:1 allocation defined by a website (randomization.com). As we will have the participant's characteristics, immediately after the randomisation the age and motor function (GMFCS/MACS) will be compared between groups; if the groups are not homogeneous, a new randomisation will be carried out. This protocol will be repeated until there is no difference between age and GMFCS amongst groups (in a maximum of first three attempts at randomisation, we always have homogeneous groups). Randomisation will be under the control of a blinded investigator who will be the only person allowed to manage the electronic security file of the randomisation to locate the individuals. (More details about this can be found in the section that follows). The investigator will be blind to the group to which the participant is allocated.

### Blinding

The participants, the researchers delivering the intervention, those performing the assessments, and the statistician will be blind to group allocation until after the data analysis. To ensure proper blinding, participants will receive codes and will be separated from the allocation process by a different investigator. The researchers responsible for applying the intervention and the outcome assessors will not know the allocation of the participants. In addition, for the blinding of the experimenter, the device to be used has a “study” mode, in which a code is inserted for each participant, so the device (DS-Stimulator Mobile, neuroConn^®^, Ilmenau, Germany) recognises and programmes the settings (active or sham). Further details about settings used in both active and sham interventions are presented in section tDCS Intervention.

### Assessment Scales and Tasks

We will use two classification systems to characterise both groups, five assessments to characterise participants and to measure improvement, and one physiological assessment; and one enjoyment scale, four visual assessments, one cognitive assessment, and two VR tasks (reaction time and coincident timing) for motor performance.

### Classification Systems for Group Characterisation

#### Manual Ability Classification System (MACS) for Children With CP

The MACS describes how children with CP use their hands to manipulate objects in daily activities, and is used for children and adolescents aged 4 to 18 years (34, 35).

The MACS has five levels. They are based on a child's ability to initiate the manipulation of age-appropriate objects alone and on the need for assistance or adaptation to perform manual activities in daily life. Levels are determined by a parent or caregiver who regularly observes the child's day-to-day functions in collaboration with a healthcare professional.

Children who are able to manipulate objects easily with maximum limitations to perform manual tasks that require speed and accuracy are classified regardless of their age as level I, and those who handle objects of lower quality and speed are classified as level II. Children at level III manipulate objects with difficulty and need help or an adapted activity, and those at level IV require continuous support and assistance and/or adapted equipment adapted to partially perform the activity. Finally, children at level V are severely compromised in manual skills and need full assistance. Given the difficulties associated with this level, it will be an excluded item in our study.

#### Gross Motor Function Classification System (GMFCS)

GMFCS is a reliable and valid standard classification system for measuring the functional abilities of children with CP (36). It describes self-initiated movement and the use of assistive devices (walkers, crutches, canes, wheelchairs and so on) for mobility during an individual's daily activities.

It uses locomotion as a key assessment and analyses the individual at five levels of locomotor performance, separated by age range from 0 to 18 years (37, 38). Thus, an individual classified as GMFCS I is able to walk without limitations. A child classified as level II may walk with limitations, where a GMFCS II operation may result in the use of wheeled mobility over long distances. A GMFCS III-graded child can usually walk with a portable mobility device indoors, but uses wheeled mobility in the community over longer distances. A GMFCS IV-rated individual may sit supported, but their own mobility is limited and they are often carried in a manual wheelchair or use motorised mobility. Children classified as GMFCS V have more severe limitations with head and trunk control, and self-mobility is only possible with an electric wheelchair (37). Considering the difficulties that are associated with level V, it will be an excluded item for our study.

### Assessments to Characterise Participants and Measure Improvement

#### Pediatric Evaluation of Disability Inventory (PEDI)

The PEDI is a standardised instrument consisting of a structured interview with the caregiver, capable of documenting the functional performance of children between 6 months and 7 years old in their daily life activities (39, 40).

This test covers three domains: self-care, mobility, and social function. The self-care scale covers food, personal hygiene, toilet use, clothing, and toilet control. The functional items of mobility provide information about transfers, walking indoors and outdoors, and use of stairs. The social function dimension reflects issues related to communication, problem solving, interaction with colleagues, amongst others.

Total scores are calculated for each scale in each domain, where each item receives a score of 0 (the child is unable to perform the activity) or 1 (the activity is part of the child's repertoire), and the sum of the items generates the score for each domain. Studies have shown that the PEDI test is valid and sufficiently reliable to be applied to children with CP in Brazil (40, 41).

#### ABILHAND-Kids

ABILHAND-Kids is a questionnaire about manual ability in self-care activities in children with upper limb involvement based on their parents' perception (42, 43).

The scale consists of 21 mainly bimanual items classified by parents as impossible, difficult, easy to complete, or unknown, defining a one-dimensional measure of manual skill in children with CP.

Scores are significantly related to school education, CP type, and gross motor function, but not to age, sex, or laterality (42, 44).

Finally, ABILHAND-Kids measures are significantly related to GMFCS levels; a higher manual skill is related to a higher gross motor function. A similar relationship between bimanual fine motor function and GMFCS levels has been found previously (45).

#### ACTIVLIM-CP

The ACTIVLIM-CP is a questionnaire for parents that measures the performance of global activity in daily activities. It has been validated for children with CP (46–48).

It includes 43 items of activities of daily living related to self-care, mobility, and domestic life, and represents a valid and reliable measure of the performance of global activity. In addition, the ACTIVLIM-CP was built based on parents' perception. They are asked to estimate the ease or difficulty their children have in performing each activity, by rating this on a three-level scale: impossible (the child is unable to perform the activity without using any other help), difficult (the child is able to perform the activity without any help, but experiences some difficulty), or easy (the child is able to perform the activity without any help and experiences no difficulty).

### Physiological Assessment (HRV)

We will use HRV to analyse autonomic nervous systems before, during, and after the intervention recovery. The analysis will follow the guidelines of the Task Force of the European Society of Cardiology and the North American Society of Pacing and Electrophysiology (49). The strap (for data collection) will be positioned on the participant's chest, and the Polar V800 (Polar Electro, Finland) heart rate receiver will be positioned next to it. HRV will be recorded after the initial assessments at rest for 10 min and during VR combined with tDCS training for 20 min. For analysis of HRV data at rest 1,000 consecutive resting rate (RR) intervals will be used, and during the tasks 256 consecutive RR intervals will be used.

Heart rate will be recorded beat by beat throughout the protocol by the Polar V800 heart rate receiver and RR intervals recorded by the monitor will be transferred to the Polar ProTrainer program, which allows HR visualisation and cardiac period extraction in the “txt.” file format.

Moderate digital filtering will be performed in the program itself, complemented with manual filtering performed in Excel software to eliminate premature ectopic beats and artefacts, and only series with more than 95% sinus beats will be included in the study (50).

HRV analysis will be performed using linear (time and frequency domain) and non-linear methods that will be analysed using Kubios HRV^®^ software (Kubios HRV v.1.1 for Windows, Biomedical Signal Analysis Group, Department of Applied Physics, University of Kuopio, Finland).

### Enjoyment Scale

An enjoyment scale using smiley faces (0 is “not fun at all;” 1 is “boring;” 2 is “a bit of fun;” 3 is “fun;” and 4 is “great fun”) will be applied after the end of the game sequences, since the motivation may be related to the motor proficiency level.

This scale was developed by Jelsma et al. (51) to evaluate how children feel when interacting with proposed non-immersive VR games. It was used in other studies using different games (52, 53).

In this study, the scale will be applied in the first and last days of the protocol to verify the children's level of satisfaction with the games presented.

### Visual Assessments

For visual evaluation the following tests will be used: the Ishirara Test and the Titmus Test.

#### Ishihara Test

The Ishihara Test (chromatic vision—Ishirara pseudoisochromatic slides) is the best known and most widely used in the world for green and red colour perception or colour blindness. It was originally created to diagnose congenital colour vision deficiencies, but it has also been shown to be effective in identifying acquired colour deficiencies (54, 55). Its application is based on the analysis of planks formed by coloured circles, with two or three shades and different sizes on a background of similar colour and structure, in which a number or maze appears in a certain colour, which should be identified by the possible bearer of the disability (56).

The boards can be classified into: demonstration boards—visible to all observers in which the figure is presented with a significant contrast brightness against the background, making chromatic sensitivity not necessary for a correct answer; and masking boards—only individuals with normal vision can see the picture in which the object is close in colour to the background (54, 56).

#### Titmus Test

The Titmus Test is a test used to assess stereoscopic vision or depth perception (the “3D view”) that is given by both eyes together and based on the principle of polarisation. It is composed of a two-sided book, and on each side are arranged figures that are projected in duplicate and horizontally disparate from each other (57).

With the use of polarised glasses, and the book positioned between 30 and 40 cm from the eyes, the participant is instructed to indicate the figures they perceive in “relief” (three-dimensional). This perception of three-dimensionality is image disparity, and is measured in arc seconds (57–59).

Quantitatively the steroscopic acuity in this test ranges from 3,000 to 40 seconds of arc, and the level of image disparity decreases as the participant is able to identify them. Therefore, the lower the numerical value in arc seconds, the greater the stereoscopic acuity (57, 59).

### Cognitive Assessment

The Wechsler Intelligence Scale for Children (WISC-IV) was developed to assess intelligence in children and adolescents. Because it assesses different intellectual aspects, WISC-IV can be used in different situations, such as psychoeducational, clinical and neuropsychological assessment, diagnosis of neurodevelopmental disorders, and psychiatric disorders (60, 61), and is indicated for the evaluation of subjects between 6 and 16 years old (62, 63).

The WISC-IV is composed of 15 subtests divided into four indices: verbal comprehension, perceptual organisation, working memory, and processing speed (61). The Intelligence Quotient is considered a global cognitive functioning index and traditionally used as a crucial measure in case-control studies in neurodevelopmental disorders (64).

### VR Task for Assessment

We will use two VR tasks to assess the participants' capacity and improvement with intervention. The tasks will be used as assessment at three points: initial assessment (D0); assessment after 5 sessions (D5); and final assessment (D11).

The assessment tasks are presented below:

#### Coincident Timing

**Figure 3:**
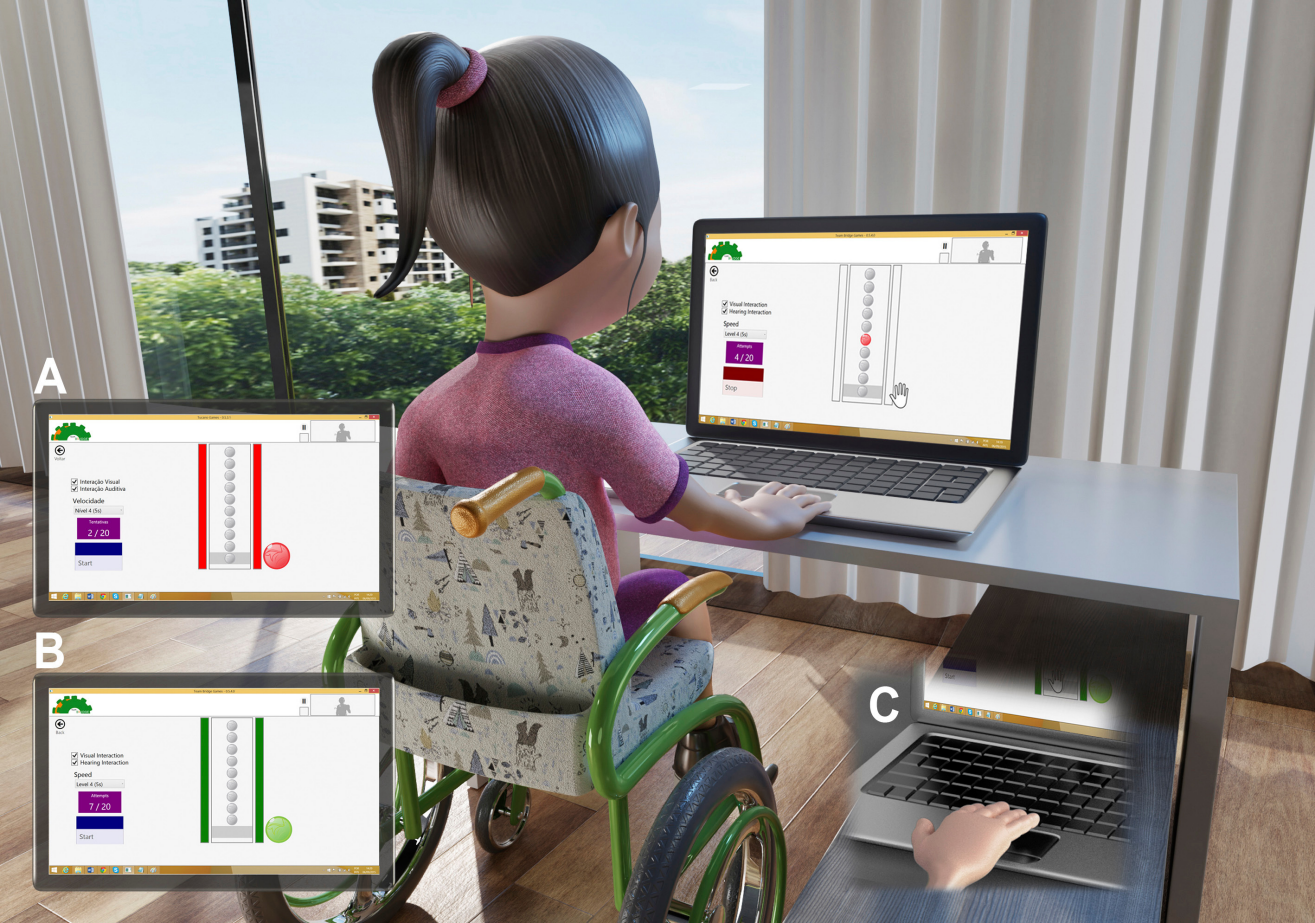
Representative design of the accomplishment of the coincident timing task. **(A)** Demonstration of error performed by the participant on each attempt (red light–unsuccessful). **(B)** Demonstration of hit performed by the participant on each attempt (green light–successful). **(C)** Demonstration of the movement on the space bar on keyboard to perform the task. Main image is about an example of the initial position of the participant.

Coincident timing is defined as the perceptual motor ability to perform a motor response in synchrony with the arrival of an external object at a given point (65, 66). This task will use non-immersive virtual coincident timing software, which displays on the computer screen (of a 15' computer) 10 (3D) spheres that light up (in red) in sequence until the last sphere—the target—is reached. The participant must rest his/her hand next to the keyboard of the computer and then press the space key at exactly the moment when the last (target) sphere lights up. The software provides immediate feedback on the success or otherwise of the task by means of different previously demonstrated sounds and colours [for more details, see (8, 17, 67, 68)]. If the participant anticipates or delays the timing of the stimulus, a red light will appear around the feedback; if he/she hits the target, a green light will appear (Figure 3).

The magnitude and direction of error of each participant in anticipating or delaying the arrival of the light is recorded by the software in milliseconds. The object is to evaluate the time difference between the participant's response and the arrival of the object at the target location (accuracy) and his/her global temporal precision and therefore his/her coincidence–anticipation ability (8, 17, 67, 69, 70).

The software is programmed to provide a unique username for each participant and the following data are stored: participant name, date of birth, sex, and the researcher's name.

#### Reaction Time

**Figure 4:**
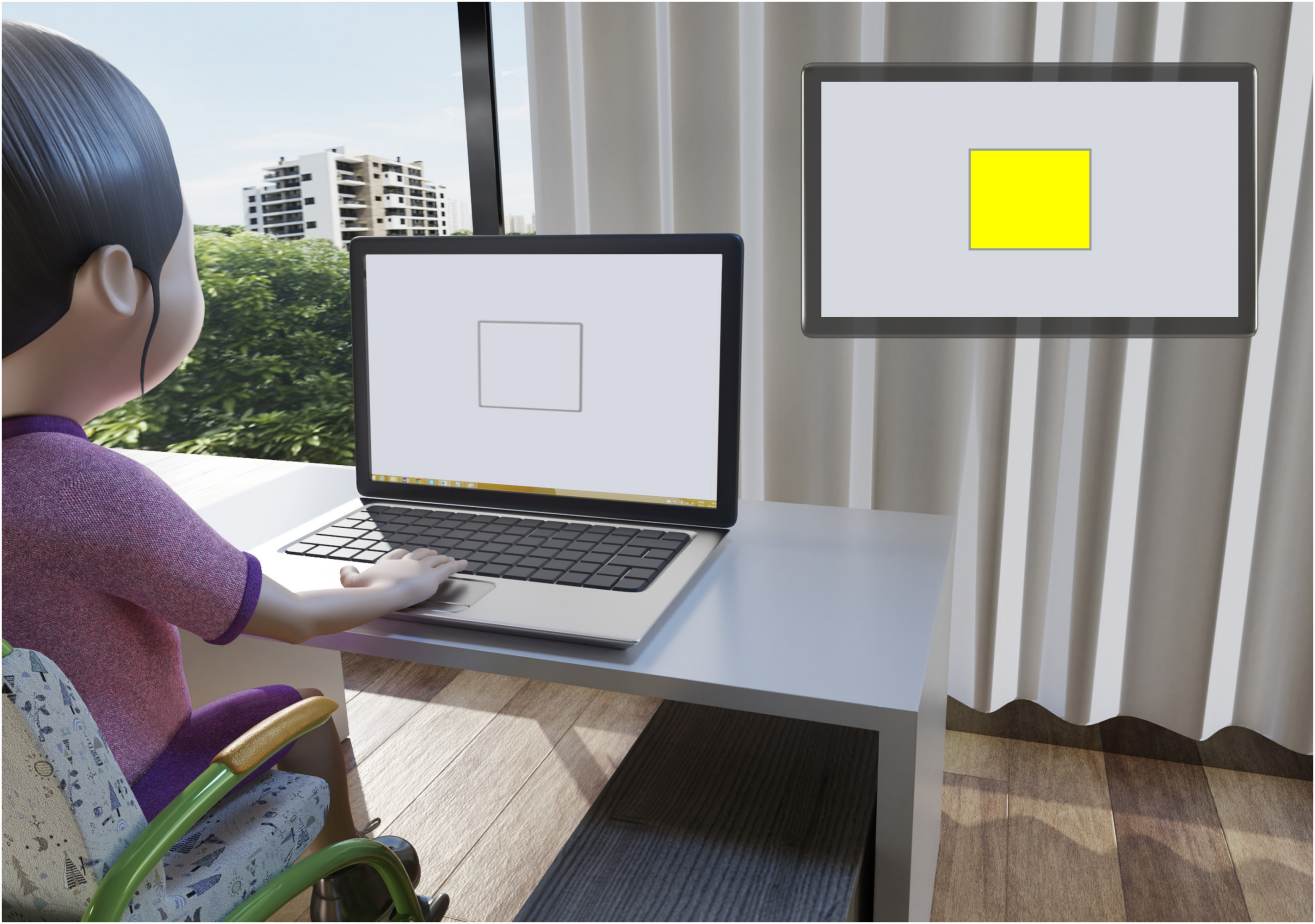
Reaction time task.

To analyse the reaction time, the software TRT_S2012 will be used [constructed and validated by (71)]. The software proposes a simple total reaction time (TRT) test, which consists of the appearance of a yellow square (parameterisable) in the centre of the monitor at predefined time intervals (ranging from 1.5 to 6.5 ms) and, when it is presented, the participant should react as quickly as possible by pressing the spacebar of the computer keyboard (Figure 4).

## Assessment Protocol

The assessment protocol will have the following sequence: the assessment scales will be undertaken with the participants' parents in a separate room (PEDI, Abilhand-Kids, and *ACTIVLIM-CP*), and GMFCS and MACS will be carried out by observacional analysis of their abilities. Also, cognitive (WISC-IV) and visual assessments (*Titmus Test and Ishihara Test)* will be made by a psychologist and a psychometrist, respectively.

Then, for the VR assessment, the coincident timing task will be carried out with a short-term motor learning protocol as used by Monteiro et al. (17), with 20 repetitions for acquisition. After 15 min of no contact with the task, the participants will perform five repetitions of the same task (for retention analysis), and five more repetitions with a speed increase (for transfer of performance analysis).

The reaction time task will be carried out with two attempts of adaptation and 10 attempts for analysis, as used by Crocetta et al. (71).

The HRV will be assessed by 20 min of rest seated in a comfortable chair or their own wheelchair, as used by Alvarez et al. (72).

The assessment part of the protocol will take around 1 h and 30 min in total (inferential analysis method follows in the item “Data analysis”).

## Intervention

All participants will attend the assigned tDCS and VR intervention as follows: there will be 20 sessions over 4 weeks with tDCS and non-immersive VR tasks, 10 of which will involve active tDCS combined with VR tasks and 10 will involve sham tDCS combined with VR tasks, separated by a 1 month washout period. The sessions will be administered consecutively and once a day (except for weekends). The investigators will have certification to apply the tDCS in children and adolescents with CP and will have experience of the VR tasks.

### VR Intervention

During application of the active or sham tDCS, in all sessions the participants will perform tasks in a non-immersive VR environment to stimulate and verify improvement of motor performance. Thus, we will use the *Bridge Games* software tasks [for details and publication, see (68, 71)]. The software that will be used was developed by the Research Group and Technological Applications in Rehabilitation (Grupo de Pesquisa e Aplicação Tecnológica em Reabilitação–PATER) group from the School of Arts, Sciences, and Humanities of the University of São Paulo (EACH-USP) (12).

The two tasks that will be used are presented below.

#### MoveHero

**Figure 5:**
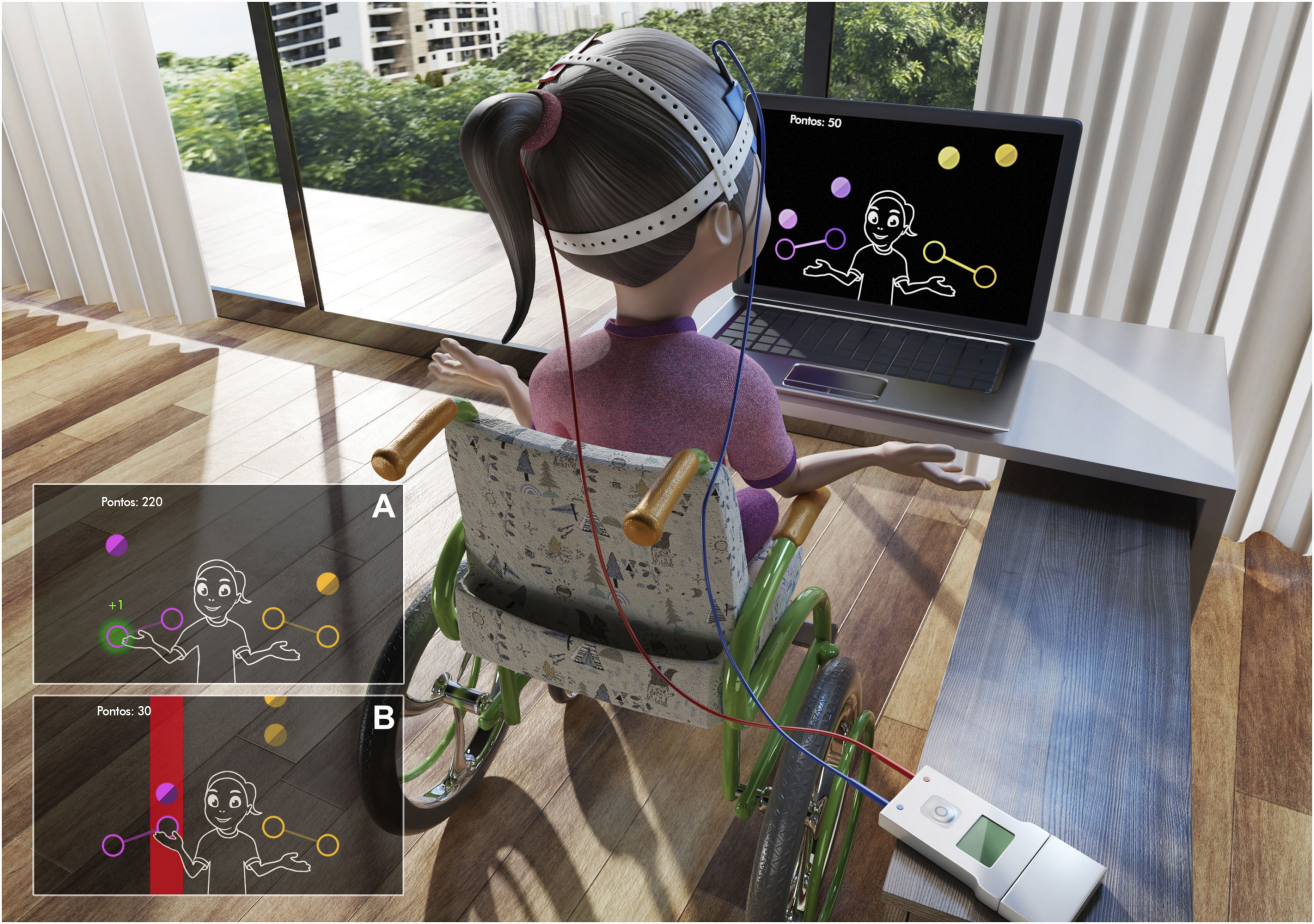
Representative design of the *MoveHero* software performed during tDCS intervention. **(A)** Demonstration of hit performed by the participant (green light). **(B)** Error performed by the participant (red bar).

*MoveHero*, as presented by Martins et al. (68), is a game that displays falling spheres in four imaginary columns on the computer screen, with a musical rhythm selected by the researcher. This is also considered a coincident timing task; the action is to react (using the upper limbs) and not let the balls pass from the fixed targets. The spheres should only be intercepted when they reach the targets allocated in parallel (at two height levels), two on the left (left position targets A and B) and two on the right of the participant (right position targets C and D), as shown in Figure 5. The virtual contact is performed by the avatar of the individual, i.e., a representation of the individual appears on the computer screen. The individual moves their arms and trunk (only if they can move the trunk) in front of the webcam to coincide with the moment the ball touches the target. The individual is positioned at a distance of 1.5 m metres from the computer monitor and waits for the balls (which fall randomly on each target) to drop. The avatar's hand should reach the target sphere along with the arrival of the ball, and the game offers feedback on correctness and error by means of changing the spheres' colour (green for correct and a red line for error).

#### Moviletrando

**Figure 6:**
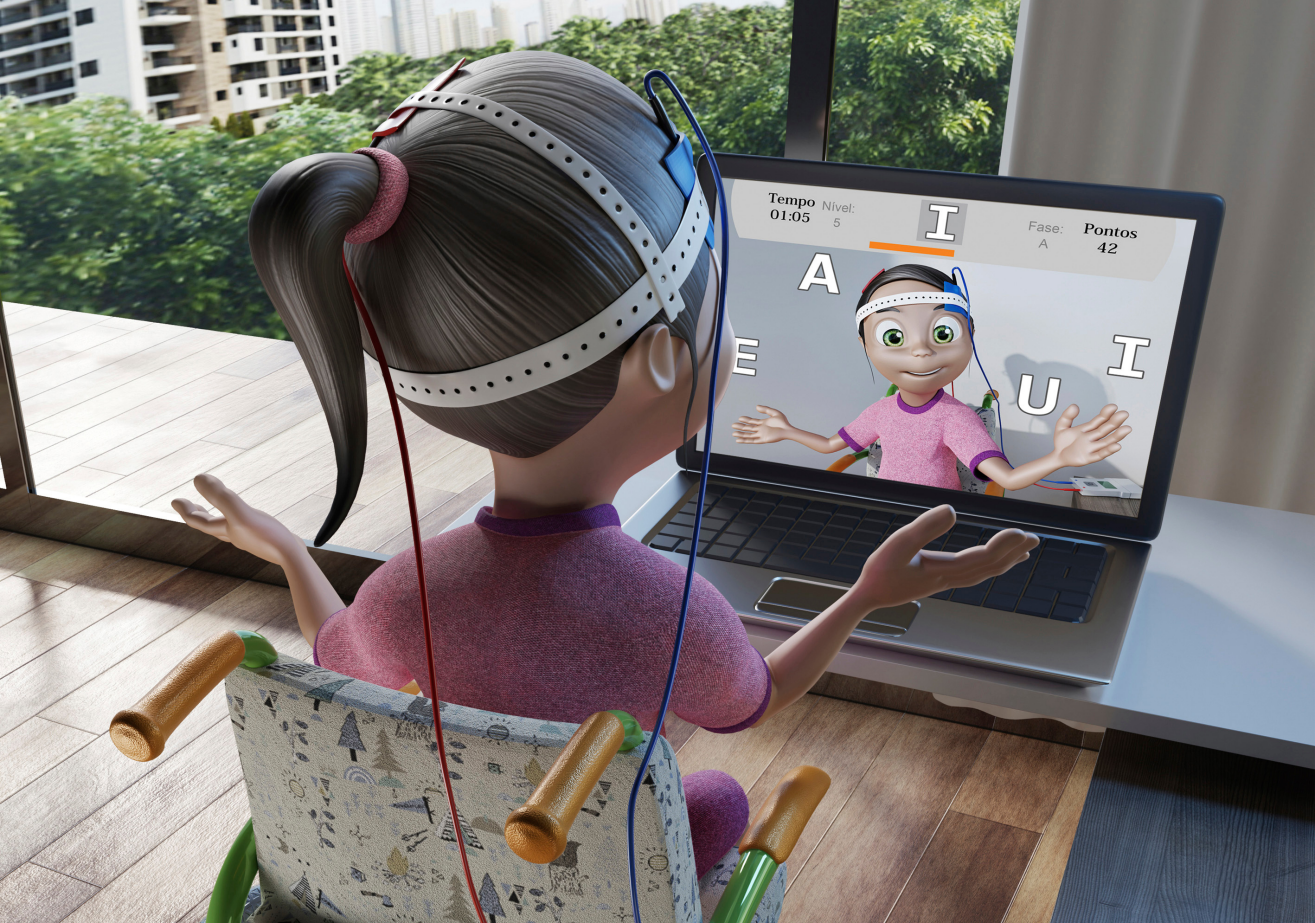
Representative design of the *Moviletrando* software performed during tDCS intervention.

The computer game *MoviLetrando* was developed at the Laboratory for Research on Visual Applications in the State University of Santa Catarina, Brazil (73, 74). It has been used in different studies [see (75, 76)]. The game uses the concept of projection-based VR with a webcam and creates a mirror images so that participants can see themselves on the screen (Figure 6).

As presented by Guarnieri et al. (75), *MoviLetrando* is a face-to-face learning computer program that involves interaction with virtual symbols projected on the screen: letters of the alphabet (vowels and/or consonants) and numbers (1 to 10). The software allows the therapist or education professional to control different phases that are identifiable as alphabet phases (AP) and numbers phases (NP). In each phase, the software offers various levels of difficulty (generating symbols on the left side, on the right side, or both sides; an increase/decrease in the number of symbols; an increase/decrease the size of the symbols; and an increase/decrease in the time of the exposure to symbols). For this study we chose two phases (one alphabet and one number). The game shows a symbol (an alphabet or number according to the phase) in the top middle of the screen, and the participant has to reach the same symbol, moving his/her hands in the virtual environment. The score obtained is based on whether or not participants reach a symbol, whether it is correct, and the elapsed time taken to carry out each task.

### tDCS Intervention

#### Active tDCS

The anodal active tDCS will be performed over 10 consecutive sessions per weekday (i.e., one session daily, no stimulation during the weekend) during the practice of VR games. The active tDCS will be performed with a current of 1 mA and 20 min of duration (and 30 s of ramp-up and ramp-down). The stimulation target will be the M1 area and aimed at the elbow, shoulder, and trunk of the Penfield homunculus (i.e., 10% instead of 20% laterally to CZ), choosing the more functional side of the participant (C1 or C2 areas of the International 10–20 System for EEG).

#### Sham tDCS

The sham tDCS will be performed over 10 consecutive sessions per weekday (i.e., one session daily, no stimulation during the weekend). However, the electrodes will be positioned at the same sites of the active tDCS and the device will be switched on for 30 s (ramp-up), giving the children the initial sensation of the 1 mA current, but with no stimulation administered the rest of the time (32). The current will be interrupted after 30 seconds. This sham protocol is already programmed in the device prior to data collection.

## Procedure

During the tDCS combined with VR protocol, participants will be seated comfortably in an ordinary chair or their own wheelchair, with their hands arranged over their legs and their feet resting on the floor (or on the wheelchair support). The demarcation and application of the active TDCS will then be performed in the cortical area corresponding to the C1 and C2 primary motor cortex according to the International 10–20 System for EEG (area M1), in order to reach the elbow, shoulders, and trunk.

Therefore, anodal tDCS with electrodes with 25 cm^2^, intensity of 1 mA and a density of up to 0.057 mA/cm^2^ for a period of 20 min will be used. The same active procedure setting will be used for the sham (placebo) procedure; however, the current will be interrupted after 30 s (32). This configuration will ensure that the electrical stimulus is interrupted before generating considerable stimuli, while the other characteristics of the intervention will be maintained. After each session the participants will be questioned about the presence of any adverse effects. The device used will be the DS-Stimulator Mobile from NeuroConn, which allows blindness of the subjects of the research and the experimenters.

After 5 min of stimulation, the individuals will perform the VR training. The protocol will count on the execution of the following sequence of games: *MoveHero* for 5 min and *Moviletrando* for another 5 min. The participants will have the rest of the time (5 min) with tDCS (sham or active) only. The training time will take 20 min in total. This kind of protocol was used by previous authors who used tDCS (22). The method of inferential analysis follows is described in the “Analysis of the data” section of this paper.

## Primary Outcome

To evaluate the effect of the combined therapy of virtual reality and tDCS in the upper-limb and trunk motor area on M1 (C1 or C2) of individuals with CP, and to check the possibility of generating motor gains in individuals with CP, we will observe the change from baseline motor values provided by different scales and VR tasks.

### Assessments for Primary Outcome

The VR task will be used to assess the participants' motor abilities (accuracy, precision, and trend of anticipation or delay in movement) during each intervention. Also, we will use coincident timing and reaction time tasks, ABILHAND-Kids, ACTIVLIM-CP, and visual and cognitive assessments to characterise the group and to find influences on motor improvement by using correlation tests. These tests will be carried out at three stages: initial assessment (D0); assessment after five interventions (D5) and final assessment (D11) (Figure 1).

## Secondary Outcome

We will observe changes in the ANS after active and sham tDCS combined with VR tasks in children and adolescents with CP.

### Assessments for Secondary Outcome

In addition to the motor tests, the HRV analysis will be verified throughout the intervention. Some studies point to ANS alteration, with a reduction in HRV in individuals with CP (77, 78). HRV represents the autonomic function, and short-term HRV measurement has been used to evaluate sympathetic and parasympathetic heart rate modulation. Therefore, because of the ease of evaluation (non-invasively, through a chest strip) and because of its high clinical relevance, it is important to evaluate HRV before and during the intervention with tDCS and VR, since some studies indicate improvement of the autonomic balance after VR tasks in individuals with Duchenne muscular dystrophy (72) and post-stroke (79). In the case of CP, some studies had identified low HRV in foetuses that would later be given a CP diagnosis (80), at rest and during postural change (81), and submaximal tests (78). Low HRV is often an indicator of abnormal and insufficient ANS adaptation, which may indicate the presence of physiological malfunction in the individual (50) and is associated with an increased risk of cardiac events (6).

## Statistical Analysis

For the coincident timing task for the inferential analysis of the initial tasks (transversal) and the longitudinal protocol with tDCS and HRV as dependent variables, the error measures (constant, absolute, and variable errors) will be considered (time in milliseconds). If the data meet the assumptions for the use of parametric analysis, ANOVA will be performed to identify intra and inter-group differences. These, if any, will be detected by the *post hoc* Tukey-HSD test. If the normality assumptions are not met, non-parametric analyses will be undertaken to identify and locate the differences: a Friedman and *post hoc* Wilcoxon test (for within groups) and a Kruskal–Wallis and *post hoc* Mann–Whitney *U*-test (between groups). For the between-groups analysis of HRV indices, MANOVA will be used, with repeated measures for within groups analyses (for evaluations and follow-up) or Mann–Whitney for intergroup analyses and Friedman for intragroup analyses. A significance level of 0.05 (5%) will be defined; all intervals constructed throughout the work will be 95% statistical confidence. The statistical program used will be SPSS (Statistical Package for Social Sciences), version 26.0.

## Discussion

Although treatment with tDCS is feasible and effective, further studies with individuals with CP are essential for a better understanding of the motor and autonomic effects of treatment with VR associated with tDCS for clinical practice. Therefore, we organised this study to analyse the influence of combined therapy of VR and tDCS for children and adolescents with CP, with VR tasks for upper limb and trunk movements. As outcome measures, we chose different upper limb functional assessment scales, computer tasks for the analysis of reaction time and anticipatory timing, as well as physiological analyses such as heart rate variability (HRV). Considering our hypothesis, supported by previous studies using similar tasks (8, 68, 70, 71), we speculate that all individuals with CP will show an improvement in performance during therapy sections and will maintain this improvement in follow-up assessments (15 and 30 days). We also hypothesise that the individuals using active tDCS with present better results.

The results from this study can positively influence the rehabilitation programs and provide answers to four important topics:

1. Safety. The tDCS and VR are safe non-invasive techniques according to current knowledge (24, 82). Two systematic reviews of tDCS in children with CP (83) and paediatric motor disorders (22) found that a large number of individuals have been involved in studies with tDCS since 2014 and noted a small number of adverse effects such as erythematous rash, mild skin burn, redness, and tingling of the skin. The use of Virtual reality presented some adverse symptomatology (especially with immersive VR and commercial games) such as nausea, dizziness, disorientation, frustration for the failure of the interface to detect movement or actions, and difficulty with hand-held interfaces, mainly in positioning users with movement and postural impairments (84). Thus, to avoid adverse effects and risks to the individual, the tDCS parameters used in the present trial will be within the safety limits described in the methods, and to prevent the adverse VR effects, we will use a game developed especially for individuals with disabilities with the use of non-immersive VR task.
2. Use of non-commercial games. Despite promising studies in the literature using commercial games (85), an important question is the potential and future use of customised serious games–defined as a game developed for a specific target (84). Commercial games are designed for entertainment rather than rehabilitation and require high cognitive and motor performance, which makes them unsuitable for patients with restrictions on mobility. In contrast, studies using games specifically developed for rehabilitation have presented interesting results (71). Thus, for the present study, we selected a serious game that provided an engaging task, specially designed and created to effectively capture the performance of individuals with disabilities and provide a report of their performance (68).
3. Combined intervention for different levels of motor impairment. There is a significant gap in knowledge about the benefits of combining different technologies for the rehabilitation of upper limbs, in order to increase accuracy of movement, reaction time, coincident timing and physical activity for children and adolescents with CP with different levels of gross motor function. Thus, we hope to contribute knowledge for clinical practice by examining the effects of a combined intervention on gross motor functions in individuals classified as GMFCS I-IV.
4. Autonomic nervous system. In addition, there is another gap in knowledge concerning the adaptation of the autonomic nervous system (assessed through heart rate variability–HRV) to a combined therapy of VR and tDCS, by measuring HRV before, during, and after this intervention. Heart Rate Variability is a well-known risk marker for chronic disorders and reflects the control of autonomic nervous system in the sinus node in different health conditions, physical activity levels and exercise (70, 72, 86). HRV is impaired in individuals with CP, with a sympathetic predominance, which leads to less adaptation to physical demands (78). It characterises a cardiovascular neural profile that can lead to negative clinical results (87), such as increased cardiac arrhythmias and cardiovascular mortality (88), in addition to the risk of sudden death (86, 89), and there is evidence that maintained exercise is a feasible option to decrease cardiovascular risk in persons with sympathetic compromise (90).

Thus, we believe that the results of this study will provide scientific support for the use of combined tDCS and VR therapy in individuals with CP to improve motor skills, functionality, and the autonomic nervous system.

## Trials Status

Participant recruitment started in December 2019 and is expected to end in July 2020. Study completion is estimated by October 2020.

## Ethics Statement

The studies involving human participants were reviewed and approved by Ethical Committee of the University of São Paulo, under the number CAAE: 99577318.0.0000.5390. Written informed consent to participate in this study was provided by the participants' legal guardian/next of kin.

## Author Contributions

TS designed the study, drafted the article, collected the patient data, is going to perform the statistical analyses, and interpret the data. AF collected patient data and drafted the article. TR, AL, BS, CA, AS, ÍM, and DR-P collected the patient data and revised the manuscript. BO-F, RS, and MA provided assistance on patient data collection and revised the manuscript. JC and HD revised the manuscript critically for intellectual content. CM coordinated the study, drafted the article, and revised the manuscript critically for intellectual content. All authors read and approved the final manuscript.

## Conflict of Interest

The authors declare that the research was conducted in the absence of any commercial or financial relationships that could be construed as a potential conflict of interest.

## Abbreviations

tDCS: transcranial direct current stimulation
VR: virtual reality
CP: cerebral palsy
mA: milliampere
HRV: heart rate variability
GMFCS: gross motor function classification system
MACS: manual ability classification system
PEDI: pediatric evaluation of disability inventory
WISC: wechsler intelligence scale for children
TRT: total reaction time.

## References

[1] Rosenbaum P Paneth N Leviton A Goldstein M Bax M Damiano D A report: the definition and classification of cerebral palsy. Dev Med Child Neurol. (2007) 49:8–14. 10.1111/j.1469-8749.2007.tb12610.x 17370477

[2] Pontén E. Contracture formation in the upper limb in cerebral palsy starts early. Dev Med Child Neurol. (2019) 61:117–8. 10.1111/dmcn.14047 30246329

[3] Sarcher A Brochard S Hug F Letellier G Raison M Perrouin-Verbe B. Patterns of upper limb muscle activation in children with unilateral spastic cerebral palsy: variability and detection of deviations. Clin Biomech. (2018) 59:85–93. 10.1016/j.clinbiomech.2018.09.005 30216783

[4] Gabis LV Tsubary NM Leon O Ashkenasi A Shefer S. Assessment of abilities and comorbidities in children with cerebral palsy. J Child Neurol. (2015) 30:1640–5. 10.1177/0883073815576792 25855688

[5] Heyn PC Tagawa A Pan Z Thomas S Carollo JJ. Prevalence of metabolic syndrome and cardiovascular disease risk factors in adults with cerebral palsy. Dev Med Child Neurol. (2019) 61:1–7. 10.1111/dmcn.14148 30663044

[6] Katz-Leurer M Amichai T. Heart rate variability in children with cerebral palsy. Dev Med Child Neurol. (2019) 61:730–1. 10.1111/dmcn.14095 31049945

[7] Anaby D Korner-Bitensky N Steven E Tremblay S Snider L Avery L. Current rehabilitation practices for children with cerebral palsy: focus and gaps. Phys Occup Ther Pediatr. (2017) 37:1–15. 10.3109/01942638.2015.1126880 26865220

[8] Monteiro CBM Massetti T Silva TD Kamp J Abreu LC Leone C. Transfer of motor learning from virtual to natural environments in individuals with cerebral palsy. Res Dev Disabil. (2014) 35:2430–7. 10.1016/j.ridd.2014.06.006 24981192

[9] Wuang Y Chiang C Su C Wang C. Effectiveness of virtual reality using wii gaming technology in children with down syndrome. Res Dev Disabil. (2011) 32:312–21. 10.1016/j.ridd.2010.10.002 21071171

[10] Novak I Morgan C Fahey M Finch-Edmondson M Galea C Hines A. State of the evidence traffic lights 2019: systematic review of interventions for preventing and treating children with cerebral palsy. Curr Neurol Neurosci Rep. (2020) 20:1–21. 10.1007/s11910-020-1022-z 32086598 PMC7035308

[11] Muszkat D Polanczyk GV Dias TG Brunoni AR. Transcranial direct current stimulation in child and adolescent psychiatry. J Child Adolesc Psychopharmacol. (2016) 26:590–7. 10.1089/cap.2015.0172 27027666

[12] Grecco LAC Duarte NAC Mendonça ME Galli M Fregni F Oliveira CS Effects of anodal transcranial direct current stimulation combined with virtual reality for improving gait in children with spastic diparetic cerebral palsy: a pilot, randomized, controlled, double-blind, clinical trial. Clin Rehabil. (2015) 29:1212–23. 10.1177/0269215514566997 25604912

[13] Ji Y Ji Y Sun B. Effect of acupuncture combined with repetitive transcranial magnetic stimulation on motor function and cerebral hemodynamics in children with spastic cerebral palsy with spleen-kidney deficiency. Zhen Ci Yan Jiu. (2019) 44:757–61. 31657167 10.13702/j.1000-0607.190154

[14] Kara OK Yardimci BN Sahin S Orhan C Livanelioglu A Soylu AR. Combined effects of mirror therapy and exercises on the upper extremities in children with unilateral cerebral palsy: a randomized controlled trial. Dev Neurorehabil. (2019) 1:253–64. 10.1080/17518423.2019.1662853 31514564

[15] Lidman G Nachemson A Peny-Dahlstrand M Himmelmann KME. Long-term effects of repeated botulinum neurotoxin A, bimanual training, and splinting in young children with cerebral palsy. Dev Med Child Neurol. (2020) 62:1–7. 10.1111/dmcn.14298 31225647

[16] Bohil CJ Alicea B Biocca FA. Virtual reality in neuroscience research and therapy. Nat Rev Neurosci. (2011) 12:752–62. 10.1038/nrn3122 22048061

[17] Monteiro CBM Silva TD Abreu LC Fregni F Araujo LV Ferreira FHIB. Short-term motor learning through nonimmersive virtual reality task in individuals with down syndrome. BMC Neurol. (2017) 17:1–8. 10.1186/s12883-017-0852-z 28410583 PMC5391542

[18] Hsu T. Effects of Wii Fit^®^ balance game training on the balance ability of students with intellectualdisabilities. J Phys Ther Sci. (2016) 28:1422–6. 10.1589/jpts.28.1422 27313343 PMC4905882

[19] Brok WLJE Sterkenburg PS Self-controlled technologies to support skill attainment in persons with an autism spectrum disorder and/or an intellectual disability: a systematic literature review. Disabil Rehabil Assist Technol. (2015) 10:1–10. 10.3109/17483107.2014.921248 24848443

[20] Gelsomini M Garzotto F Montesano D Occhiuto D. Wildcard: a wearable virtual reality storytelling tool for children with intellectual developmental disability. Eng Med Biol Soc. (2016) 2016:5188–91. 10.1109/EMBC.2016.7591896 28269433

[21] Kang E Kim D Paik N. Transcranial direct current stimulation of the left pré-frontal córtex improves attention in patients with traumatic brain injury: a pilot study. J Rehabil Med. (2012) 44:346–50. 10.2340/16501977-0947 22434324

[22] Saleem GT Crasta JE Slomine BS Cantarero GL Suskauer SJ. Transcranial direct current stimulation in pediatric motor disorders: a systematic review and meta-analysis. Arch Phys Med Rehabil. (2019) 100:1–15. 10.1016/j.apmr.2018.10.011 30414398 PMC7927962

[23] Horvath JC Vogrin SJ Carter O Cook MJ Forte JD. Effects of a common transcranial direct current stimulation (tDCS) protocol on motor evoked potentials found to be highly variable within individuals over 9 testing sessions. Exp Brain Res. (2016) 234:2629–42. 10.1007/s00221-016-4667-8 27150317

[24] Nitsche MA Liebetanz D Lang N Antal A Tergau F Paulus W. Safety criteria for transcranial direct current stimulation (tDCS) in humans. Clin Neurophysiol. (2003) 114:2220–2. 10.1016/S1388-2457(03)00235-9 14580622

[25] Spampinato DA Satar Z Rothwell JC. Combining reward and M1 transcranial direct current stimulation enhances the retention of newly learnt sensorimotor mappings. Brain Stimul. (2019) 12:1205–12. 10.1016/j.brs.2019.05.015 31133478 PMC6709642

[26] O'Brien AT Bertolucci F Torrealba-Acosta G Huerta R Fregni F Thibaut A. Non-invasive brain stimulation for fine motor improvement after stroke: a meta-analysis. Eur J Neurol. (2018) 25:1017–26. 10.1111/ene.13643 29744999

[27] Makovac E Thayer JF Ottaviani C. A meta-analysis of non-invasive brain stimulation and autonomic functioning: implications for brain-heart pathways to cardiovascular disease. Neurosci Biobehav Rev. (2017) 74:330–41. 10.1016/j.neubiorev.2016.05.001 27185286

[28] Lazzari RD Politti F Santos CA Dumont AJL Rezende FL Grecco LAC. Effect of a single session of transcranial direct-current stimulation combined with virtual reality training on the balance of children with cerebral palsy: a randomized, controlled, double- blind trial. J Phys Ther Sci. (2015) 27:763–8. 10.1589/jpts.27.763 25931726 PMC4395710

[29] Lazzari RD Politti F Belina SF Grecco LAC Santos CA Dumont AJL. Effect of transcranial direct current stimulation combined with virtual reality training on balance in children with cerebral palsy: a randomized, controlled, double-blind, clinical trial. J Mot Behav. (2017) 49:329–36. 10.1080/00222895.2016.1204266 27644454

[30] Chan A Tetzlaff JM Gøtzsche PC Atman DG Mann H Berlin JA. SPIRIT 2013 explanation and elaboration: guidance for protocols of clinical trials. BMJ. (2013) 346:1–42 10.1136/bmj.e7586 23303884 PMC3541470

[31] Biabani M Farrell M Zoghi M Egan G Jaberzadeh S. Crossover design in transcranial direct current stimulation studies on motor learning: potential pitfalls and difficulties in interpretation of findings. Rev Neurosci. (2018) 29:463–73. 10.1515/revneuro-2017-0056 29232195

[32] Duarte NAC Grecco LAC Galli M Fregni F Oliveira CS. Effect of transcranial direct-current stimulation combined with treadmill training on balance and functional performance in children with cerebral palsy: a double-blind randomized controlled trial. PLoS ONE. (2014) 9:e105777. 10.1371/journal.pone.0105777 25171216 PMC4149519

[33] Massetti T Crocetta TB Guarnieri R Silva TD Leal AF Voos MC. A didactic approach to presenting verbal and visual information to children participating in research protocols: the comic book informed assent. Clinics. (2018) 73:207–12. 10.6061/clinics/2018/e207 30156595 PMC6104506

[34] Eliasson A Krumlinde-Sundholm L Rösblad B Beckung E Arner M Ohrvall A. The Manual Ability Classification System (MACS) for children with cerebral palsy: scale development and evidence of validity and reliability. Dev Med Child Neurol. (2006) 48:549–54. 10.1111/j.1469-8749.2006.tb01313.x 16780622

[35] Morris C Kurinczuk JJ Fitzpatrick R Rosenbaum PL. Reliability of the manual ability classification system for children with cerebral palsy. Dev Med Child Neurol. (2006) 48:950–3. 10.1111/j.1469-8749.2006.tb01264.x 17109781

[36] Bodkin AW Robinson C Perales FP. Reliability and validity of the gross motor function classification system for cerebral palsy. Pediatr Phys Ther. (2003) 15:247–52. 10.1097/01.PEP.0000096384.19136.02 17057460

[37] Palisano R Rosenbaum P Walter S Russel D Wood E Galuppi B. Development and reliability of a system to classify gross motor function in children with cerebral palsy. Dev Med Child Neurol. (1997) 39:214–23. 10.1111/j.1469-8749.1997.tb07414.x 9183258

[38] Rosenbaum PL Palisano RJ Bartlett DJ Galuppi BE Russel DJ. Development of the gross motor function classification system for cerebral palsy. Dev Med Child Neurol. (2008) 50:249–53. 10.1111/j.1469-8749.2008.02045.x 18318732

[39] Monteiro CBM Savelsbergh GJP Smorenburg AP Graciani Z Torriani-Pasin C Abreu LC. Quantification of functional abilities in Rett syndrome: a comparison between stages III and IV. Neuropsychiatr Dis Treat. (2014) 10:1213–22. 10.2147/NDT.S57333 25061307 PMC4086772

[40] Mancini M Inventário de Avaliação Pediátrica de Incapacidade (PEDI): manual da versão brasileira. Belo Horizonte: UFMG (2005).

[41] Castro C Batistela F Martini G Fonseca G Montesanti L Oliveira MC Correlação da função motora e o desempenho funcional nas atividades de auto-cuidado em grupo de crianças portadoras de paralisia cerebral. Med Reabil. (2006) 25:7–11.

[42] Penta M Tesio L Arnould C Zancan A Thonnard JL. The ABILHAND questionnaire as a measure of manual ability in chronic stroke patients: rasch-based validation and relationship to upper limb impairment. Stroke. (2001) 32:1627–34. 10.1161/01.STR.32.7.1627 11441211

[43] Arnould C Penta M Renders A Thonnard J. ABILHAND-Kids: a measure of manual ability in children with cerebral palsy. Neurology. (2004) 63:1045–52. 10.1212/01.WNL.0000138423.77640.37 15452296

[44] Arnould C Penta M Thonnard J. Hand impairments and their relationship with manual ability in children with cerebral palsy. J Rehabil Med. (2007) 39:708–14. 10.2340/16501977-0111 17999009

[45] van Eck M Dallmeijer AJ van Lith IS Voorman JM Becher J. Manual ability and its relationship with daily activities in adolescents with cerebral palsy. J Rehabil Med. (2010) 42:493–8. 10.2340/16501977-0543 20544163

[46] Bleyenheuft Y Paradis J Renders A Thonnard J Arnould C. ACTIVLIM-CP a new Rasch-built measure of global activity performance for children with cerebral palsy. Res Dev Disabilities. (2017) 60:285–94. 10.1016/j.ridd.2016.10.005 28341237

[47] Paradis J Arnould C Thonnard J Houx L Pons-Becmeur C Renders A Responsiveness of the ACTIVLIM-CP questionnaire measuring global activity performance in children with cerebral palsy. Dev Med Child Neurol. (2018) 60:1–8. 10.1111/dmcn.13927 29869417

[48] Burgess A Boyd RN Ziviani J Sakzewski L. A systematic review of upper limb activity measures for 5- to 18-year-old children with bilateral cerebral palsy. Aust Occup Ther J. (2019) 66:552–67. 10.1111/1440-1630.12600 31385319

[49] European Society of Cardiology Heart rate variability: standards of measurement, physiological interpretation, and clinical use. Eur Heart J. (1996) 17:354–81. 10.1093/oxfordjournals.eurheartj.a014868 8737210

[50] Vanderlei LCM Pastre CM Hoshi RA Carvalho TD Godoy MF. Basic notions of heart rate variability and its clinical applicability. Rev Bras Cir Cardiovasc. (2009) 24:205–17. 10.1590/S0102-76382009000200018 19768301

[51] Jelsma D Geuze RH Mombarg R Smits-Engelsman BC. The impact of Wii Fit intervention on dynamic balance control in children with probable developmental coordination disorder and balance problems. Hum Movement Sci. (2014) 33:404–18. 10.1016/j.humov.2013.12.007 24444657

[52] Farhat F Hsairi I Baati H Smits-Engelsman BCM Masmoudi K Mchirgui R. The effect of a motor skills training program in the improvement of practiced and non-practiced tasks performance in children with developmental coordination disorder (DCD). Hum Movement Sci. (2016) 46:10–22. 10.1016/j.humov.2015.12.001 26703915

[53] Smits-Engelsman BCM Jelsma LD Ferguson GD. The effect of exergames on functional strength, anaerobic fitness, balance and agility in children with and without motor coordination difficulties living in low-income communities. Hum Movement Sci. (2017) 55:327–37. 10.1016/j.humov.2016.07.006 27423302

[54] Ishihara S Ishihara Instructions: Test of Color Deficiency. Tokyo: Kanehara Trading Inc. (1974)

[55] Fernandes LC Urbano LCV. Eficiência dos testes cromáticos de comparação na discromatopsia hereditária: relatos de casos. Arquivo Brasileiro de Oftalmologia. (2008) 71:585–8. 10.1590/S0004-27492008000400023 18797674

[56] Bruni LF Cruz AAV. Sentido cromático: tipos de defeitos E testes de avaliação clínica. Arq Bras Oftalmol. (2006) 69:766–75. 10.1590/S0004-27492006000500028 17187151

[57] Cunha JP Ferreira J Multifocalidade e Estereopsia. Oftalmologia. (2010) 34:465–70.

[58] Fernandes LC Safe SMS Almeida HC Respostas aos testes de estereopsia em portadores de visão subnormal. Arq Bras Oftalmol. (1998) 61:202–5. 10.5935/0004-2749.19980079

[59] Oliveira F Mucciolo C Silva LMP Soriano ES Souza CEB Junior RB. Avaliação da sensibilidade ao contraste e da estereopsia em pacientes com lente intra-ocular multifocal. Arq Bras Oftalmol. (2005) 68:439–43. 10.1590/S0004-27492005000400005 16322826

[60] Cruz M WISCII: Iescala de inteligência wechsler para crianças: manual. Avaliação Psicol. (2005) 4:199–201.

[61] Dias-Viana JL Gomes GVA Wechsler Intelligence Scale for Children (WISC): analysis of the production of Brazilian scientific articles. Psic Rev. (2019) 28:9–36. 10.23925/2594-3871.2019v28i1p9-36

[62] Figueiredo V WISC-III. in Psicodiagnóstico-V, ed Cunha J. A (Porto Alegre, RS: Artmed) (2000) 603–14.

[63] Nascimento E Figueiredo VLM WISC-III e WAIS-III: alterações nas versões originais americanas decorrentes das adaptações para uso no Brasil. Psicologia. (2002) 15:603–12. 10.1590/S0102-79722002000300014

[64] Rao VS Raman V Mysore AV. Issues related to obtaining intelligence quotient - matched controls in autism research. Indian J Psychol Med. (2015) 37:149–53. 10.4103/0253-7176.155612 25969598 PMC4418245

[65] Belisle J Accuracy, reliability and refractoriness in a coincidence anticipation task. Res Q Exerc Sport. (2013) 34:271–81. 10.1080/10671188.1963.10613234

[66] Fooken J Yeo S Pai DK Spering M. Eye movement accuracy determines natural interception strategies. J Vision. (2016) 16:1–15. 10.1167/16.14.1 27802509

[67] Bezerra IMP Crocetta TB Massetti T Silva TD Guarnieri R Junior CMM. Functional performance comparison between real and virtual tasks in older adults: a cross-sectional study. Medicine. (2018) 97:1–8. 10.1097/MD.0000000000009612 29369177 PMC5794361

[68] Martins FPA Massetti T Crocetta TB Lopes PB Silva AA Figueiredo EF. Analysis of motor performance in individuals with cerebral palsy using a non-immersive virtual reality task - a pilot study. Neuropsychiatr Dis Treat. (2019) 15:417–28. 10.2147/NDT.S184510 30787616 PMC6366350

[69] Malheiros SRP Silva TD Favero FM Abreu LC Fregni F Ribeiro DC. Computer task performance by subjects with Duchenne muscular dystrophy. Neuropsychiatric Disease Treatment. (2016) 12:41–8. 10.2147/NDT.S87735 26766911 PMC4699593

[70] Moraes ÍAP Monteiro CBM Silva TD Massetti T Crocetta TB Menezes LC. Motor learning and transfer between real and virtual environments in young people with autism spectrum disorder: a prospective randomized cross over controlled trial. Autism Res. (2020) 13:1–13. 10.1002/aur.2208 31566888

[71] Crocetta TB Araújo LV Guarnieri R Massetti T Ferreira FHIB Abreu LC Virtual reality software package for implementing motor learning and rehabilitation experiments. Virtual Reality. (2017) 22:199–209. 10.1007/s10055-017-0323-2

[72] Alvarez MPB Silva TD Favero FM Valenti VE Raimundo RD Vanderlei LCM. Autonomic modulation in duchenne muscular dystrophy during a computer task: a prospective control trial. PLoS ONE. (2017) 12: e0169633. 10.1371/journal.pone.0169633 28118369 PMC5261738

[73] Duarte NAC Grecco LAC Lazzari RD Neto HP Galli M Oliveira CS. Effect of transcranial direct current stimulation of motor cortex in cerebral palsy: a study protocol. Pediatr Phys Ther. (2018) 30:67–71. 10.1097/PEP.0000000000000467 29252842

[74] Yanovich E Ronen O The use of virtual reality in motor learning: a multiple pilot study review. Adv Phys Educ. (2015) 5:188–93. 10.4236/ape.2015.53023

[75] Guarnieri R Crocetta TB Massetti T Barbosa RTA Antão JYFL Antunes TPC. Test-retest reliability and clinical feasibility of a motion-controlled game to enhance the literacy and numeracy skills of young individuals with intellectual disability. Cyberpsychol Behav Soc Netw. (2019) 22:1–11. 10.1089/cyber.2017.0534 30346804

[76] Zangirolami-Raimundo J Raimundo RD Silva TD Andrade PE Benetti FA Paiva LS. Contrasting performance between physically active and sedentary older people playing exergames. Medicine. (2019) 98:1–8. 10.1097/MD.0000000000014213 30702574 PMC6380728

[77] Verschuren O Takken T. Aerobic capacity in children and adolescents with cerebral palsy. Res Dev Disabil. (2010) 31:1352–7. 10.1016/j.ridd.2010.07.005 20674266

[78] Amichai T Eylon S Dor-Haim H Berger I Katz-Leurer M. Cardiac autonomic system response to submaximal test in children with cerebral palsy. Pediatric Physical Therapy. (2017) 29:125–8. 10.1097/PEP.0000000000000368 28350766

[79] Sampaio LMM Subramaniam S Arena R Bhatt T. Does virtual reality-based kinect dance training paradigm improve autonomic nervous system modulation in individuals with chronic stroke? J Vasc Interv Neurol. (2016) 9:21–9. 27829967 PMC5094257

[80] Nelson KB Dambrosia JM Ting TY Grether JK. Uncertain value of electronic fetal monitoring in predicting cerebral palsy. N Engl J Med. (1996) 334:613–9. 10.1056/NEJM199603073341001 8592523

[81] Amichai T Katz-Leurer M. Heart rate variability in children with cerebral palsy: review of the literature and meta-analysis. NeuroRehabilitation. (2014) 35:113–22. 10.3233/NRE-141097 24990007

[82] Saposnik G Cohen LG Mamdani M Pooyania S Ploughman M Cheung D. Efficacy and safety of non-immersive virtual reality exercising in stroke rehabilitation (EVREST): a randomised, multicentre, single-blind, controlled trial. Lancet Neurol. (2016) 15:1019–27. 10.1016/S1474-4422(16)30121-1 27365261 PMC5108052

[83] Hamilton A Wakely L Marquez J. Transcranial direct-current stimulation on motor function in pediatric cerebral palsy: a systematic review. Pediatr Phys Ther. (2018) 30:291–301. 10.1097/PEP.0000000000000535 30199513

[84] Leal AF da Silva TD Lopes PB Bahadori S de Araújo LV da Costa MVB. The use of a task through virtual reality in cerebral palsy using two different interaction devices (concrete and abstract) - a cross-sectional randomized study. J Neuroeng Rehabil. (2020) 17:59–69. 10.1186/s12984-020-00689-z 32349752 PMC7191706

[85] Chen Y Fanchiang HD Howard A. Effectiveness of virtual reality in children with cerebral palsy: a systematic review and meta-analysis of randomized controlled trials. Phys Ther. (2018) 98:63–77. 10.1093/ptj/pzx107 29088476 PMC6692882

[86] Silva TD Crocetta TB Monteiro CBM Carll A Vanderlei LC Ferreira C. Heart rate variability and cardiopulmonary dysfunction in patients with duchenne muscular dystrophy: a systematic review. Pediatric Cardiol. (2018) 39:869–83. 10.1007/s00246-018-1881-0 29696428

[87] Dalla Vecchia L De Maria B Marinou K Sideri R Lucini A Porta A. Cardiovascular neural regulation is impaired in amyotrophic lateral sclerosis patients. A study by spectral and complexity analysis of cardiovascular oscillations. Physiol Meas. (2015) 36:659–70. 10.1088/0967-3334/36/4/659 25798998

[88] La Rovere MT Bigger JTJr Marcus FI Mortara A Schwartz PJ Baroreflex sensitivity and heart rate variability in prediction of total cardiacmortality after myocardial infarction. ATRAMI (Autonomic Tone and Reflexes After Myocardial Infarction) Investigators. Lancet. (1998) 351:478–84. 10.1016/S0140-6736(97)11144-8 9482439

[89] Pinto S Pinto I De Carvalho M. Decreased heart rate variability predicts death in amyotrophic lateral sclerosis. Muscle Nerve. (2012) 46:341–5. 10.1002/mus.23313 22907223

[90] Buker DB Oyarce CC Plaza RS. Effects of spinal cord injury in heart rate variability after acute and chronic exercise: a systematic review. Top Spinal Cord Inj Rehabil. (2018) 24:167–76. 10.1310/sci17-00028 29706761 PMC5915108

